# Effectiveness of a Digital Health Intervention Leveraging Reinforcement Learning: Results From the Diabetes and Mental Health Adaptive Notification Tracking and Evaluation (DIAMANTE) Randomized Clinical Trial

**DOI:** 10.2196/60834

**Published:** 2024-10-08

**Authors:** Adrian Aguilera, Marvyn Arévalo Avalos, Jing Xu, Bibhas Chakraborty, Caroline Figueroa, Faviola Garcia, Karina Rosales, Rosa Hernandez-Ramos, Chris Karr, Joseph Williams, Lisa Ochoa-Frongia, Urmimala Sarkar, Elad Yom-Tov, Courtney Lyles

**Affiliations:** 1 School of Social Welfare University of California Berkeley Berkeley, CA United States; 2 Department of Psychiatry and Behavioral Sciences University of California-San Francisco San Francisco, CA United States; 3 Centre for Quantitative Medicine Duke-NUS Medical School National University of Singapore Singapore Singapore; 4 Faculty of Technology, Policy, and Management Delft Technical University Delft Netherlands; 5 Action Research Center (ARC) for Health Equity Department of Medicine University of California-San Francisco San Francisco, CA United States; 6 Department of Psychological Sciences University of California Irvine Irvine, CA United States; 7 Audacious Software Chicago, IL United States; 8 Department of Computer Science University of Toronto Toronto, ON Canada; 9 Department of Medicine University of California–San Francisco San Francisco, CA United States; 10 Department of Computer Science Bar-Ilan University Ramat Gan Israel; 11 Center for Healthcare Policy and Research UC Davis Health Sacramento, CA United States; 12 Department of Public Health Sciences UC Davis School of Medicine Sacramento, CA United States

**Keywords:** digital health, physical activity, mobile phone, text messages, SMS, steps, walking, diabetes, depression, reinforcement learning, exercise, machine learning

## Abstract

**Background:**

Digital and mobile health interventions using personalization via reinforcement learning algorithms have the potential to reach large number of people to support physical activity and help manage diabetes and depression in daily life.

**Objective:**

The Diabetes and Mental Health Adaptive Notification and Tracking Evaluation (DIAMANTE) study tested whether a digital physical activity intervention using personalized text messaging via reinforcement learning algorithms could increase step counts in a diverse, multilingual sample of people with diabetes and depression symptoms.

**Methods:**

From January 2020 to June 2022, participants were recruited from 4 San Francisco, California–based public primary care clinics and through web-based platforms to participate in the 24-week randomized controlled trial. Eligibility criteria included English or Spanish language preference and a documented diagnosis of diabetes and elevated depression symptoms. The trial had 3 arms: a Control group receiving a weekly mood monitoring message, a Random messaging group receiving randomly selected feedback and motivational text messages daily, and an Adaptive messaging group receiving text messages selected by a reinforcement learning algorithm daily. Randomization was performed with a 1:1:1 allocation. The primary outcome, changes in daily step counts, was passively collected via a mobile app. The primary analysis assessed changes in daily step count using a linear mixed-effects model. An a priori subanalysis compared the primary step count outcome within recruitment samples.

**Results:**

In total, 168 participants were analyzed, including those with 24% (40/168) Spanish language preference and 37.5% (63/168) from clinic-based recruitment. The results of the linear mixed-effects model indicated that participants in the Adaptive arm cumulatively gained an average of 3.6 steps each day (95% CI 2.45-4.78; *P*<.001) over the 24-week intervention (average of 608 total steps), whereas both the Control and Random arm participants had significantly decreased rates of change. Postintervention estimates suggest that participants in the Adaptive messaging arm showed a significant step count increase of 19% (606/3197; *P*<.001), in contrast to 1.6% (59/3698) and 3.9% (136/3480) step count increase in the Random and Control arms, respectively. Intervention effectiveness differences were observed between participants recruited from the San Francisco clinics and those recruited via web-based platforms, with the significant step count trend persisting across both samples for participants in the Adaptive group.

**Conclusions:**

Our study supports the use of reinforcement learning algorithms for personalizing text messaging interventions to increase physical activity in a diverse sample of people with diabetes and depression. It is the first to test this approach in a large, diverse, and multilingual sample.

**Trial Registration:**

ClinicalTrials.gov NCT03490253; https://clinicaltrials.gov/study/NCT03490253

**International Registered Report Identifier (IRRID):**

RR2-10.1136/bmjopen-2019-034723

## Introduction

Noncommunicable diseases such as type 2 diabetes and depression are significant public health problems individually and interact to worsen outcomes for each other [1]. Depression is associated with unhealthy behaviors such as physical inactivity and poor dietary habits, which are also risk factors for the development of type 2 diabetes [2]. The psychosocial burden of living with a chronic illness such as diabetes can contribute to the development and worsening of depression—leading to a cycle of multiplicative negative health effects. Diabetes and depression exhibit racial and socioeconomic disparities stemming from known barriers from the individual to the structural levels, such as the stressors due to discrimination and poverty, difficulty in accessing care related to language barriers, competing health demands, and beyond [3,4].

Physical activity is a core intervention for both type 2 diabetes and depression, as both are associated with low physical activity levels [5,6]. Increased physical activity has positive effects on both conditions, including improved glycemic control in diabetes and reduced symptoms of depression [7]. Behavioral activation interventions that incorporate physical activity as a core element are among the most effective for treating depression, relative to other behavioral and psychological approaches [8,9]. Targeting physical activity is thus an efficient method of improving outcomes in both diabetes and depression.

Digital and mobile health interventions have the potential to reach a large number of people to support physical activity and help manage diabetes and depression in daily life, with a large research base using digital technology to improve and personalize health behavior programs [10,11]. Machine learning algorithms can help personalize and optimize interventions by tailoring them to the individual’s specific needs, preferences, and capabilities. Reinforcement learning algorithms, in particular, have the potential to create adaptive and dynamic interventions focused on a goal or reward that can adjust based on an individual’s responses and behaviors [12]. In previous work these algorithms increased physical activity among patients with diabetes [13]. However, novel data science methods such as personalization via reinforcement learning algorithms typically do not include data from marginalized populations (ie, ethnic and racialized minoritized populations and those from low-income backgrounds) [14]. It is crucial that digital platforms and newer algorithms are built with diverse populations to increase their generalizability and effectiveness, particularly to make an impact on entrenched health inequities.

The Diabetes and Mental Health Adaptive Notification Tracking and Evaluation (DIAMANTE) study aimed to address these gaps by developing and evaluating a reinforcement learning-based digital health app co-designed with English- and Spanish-speaking adults with comorbid diabetes and depression symptoms. The app used reinforcement learning algorithms to tailor daily text messages based on feedback type, motivation type, and message timing. The algorithm training data included demographic (eg, age), clinical data (eg, baseline depression scores), and contextual data (eg, timing and category of messages previously sent) [15]. Here, we present the efficacy of the DIAMANTE intervention, in a 3-arm randomized trial, on daily step count outcomes.

## Methods

### Study Design

We conducted a randomized controlled trial (RCT) with 3 arms from January 2020 to June 2022. We used the Standard Protocol Items: Recommendations for Interventional Trials checklist for reporting our findings [16]. A study protocol and a correction to the protocol outlining changes in recruitment approach were published in 2020 and 2023, respectively [15,17].

### Participants

RCT recruitment occurred on 2 tracks. Clinic-based patient recruitment at the San Francisco Health Network (SFHN, the public health care delivery system for the city and county of San Francisco, California) was our first method of enrolling study participants. These clinic-based strategies were significantly impacted by the COVID-19 pandemic. Prior to March 2020 and after California’s reopening in June 2021, patients were recruited by direct provider referrals, with in-person clinic recruitment timed to eligible patient visits. To identify potentially in-person eligible patients, we asked for permission from known providers to access patient lists and then reviewed and identified patients with documented electronic health records diagnosis of diabetes and elevated depression symptoms (depression diagnosis or Patient Health Questionnaire (PHQ-8) score of 5 or higher in the past 5-year period) and whether they were adults aged 18 years or older. Eligible patients were required to use text messaging and own a smartphone in order to download the pedometer app into their phone. Patients were excluded if they were unable to walk or were pregnant at the time of the study. Researchers then contacted patients directly (via in-person visits and via phone calls) to determine interest in joining the DIAMANTE study and review study eligibility criteria. Interested individuals were invited for a study visit that included obtaining informed consent and completing a baseline survey. All patients were offered assistance in downloading the DIAMANTE app onto their phone and sent test text messages back to our system. The researcher established a baseline plan for physical activity goals with the patient (ie, average 4000 steps daily) and instructed patients to have the app open at all times. To replicate real-world conditions and increase generalizability, participants were not provided with any instructions about how much, when, or where in their body to carry their phones. All participants were invited back at 24-weeks to complete a follow-up survey when the active intervention period ended.

We added a second web-based recruitment strategy during the COVID-19 pandemic, given that in-clinic recruitment was not possible for many months during the active enrollment period for our RCT. Specifically, between March 2020 and October 2021, we recruited through social media advertisements via advertisements posted on Facebook, Google Ads, and Craigslist. Potential participants completed a screening survey to assess eligibility (ie, self-reported diabetes diagnosis and PHQ-8). In addition to the exclusion criteria, if participants were unable to walk or currently pregnant, we also excluded those who did not have an eligible phone number, were outside of the United States, or those who failed the CAPTCHA requirements to verify their identity on the web. Web-based participants were enrolled remotely, offering Zoom (Zoom Video Communications, Inc) meeting and phone assistance to complete all enrollment procedures (eg, onboarding, downloading the app, and setting a step count goal) as needed. All informed consent and survey data collection matched our in-clinic recruitment procedures.

### Ethical Considerations

The study was approved by the University of California San Francisco (UCSF) institutional review board (17-22608). The project coordinator and research assistants were responsible for managing patient data collection. Self-reported participant data were stored on REDCap (Research Electronic Data Capture; Vanderbilt University), surveys were stored on UCSF Qualtrics, and daily step count and text messaging data were stored on the HealthySMS platform [18]. To maintain patient privacy and confidentiality only institutional review board–approved research staff had access to the data collection platforms. Data were downloaded from their respective servers and stored in a secure UCSF Box Folder. All participants gave written or electronic informed consent in the language of their choice, English or Spanish, prior to participating in the study. Patients received a compensation of US $40 in cash for participating in the baseline questionnaire and an additional US $70 in cash for the 24-week follow-up questionnaire.

### Randomization

Randomization was performed with block randomization (block size 3) and a 1:1:1 allocation into arms (adaptive messaging, random messaging, and app only), stratified by patient language preference (English vs Spanish). Upon sign-up, participants were automatically randomized via a randomization table in the HealthySMS system (developed by author CK) into one of the study conditions after they were onboarded into the study, thereby ensuring allocation concealment. Patients were informed of the nature of the app and frequency of the messages they would be receiving. The necessity of these steps made it unfeasible to fully blind participants or research staff; however, the final data analyses were completely blinded.

### Procedures

In brief, the DIAMANTE study compared the effectiveness of different text messaging strategies on physical activity (measured by step counts on participants’ smartphones) over an active 24-weeks intervention period. We created a custom mobile phone app titled “DIAMANTE” developed by Audacious Software for this study. This application passively tracks step counts by pooling from Google Fit, Apple HealthKit, or the built-in pedometer on patients’ phones. We used a text messaging platform HealthySMS, previously developed by Dr Aguilera and Audacious Software, to integrate with the DIAMANTE app and send text messages by intervention arm. The DIAMANTE app only needed to be installed once and then remain open consistently. The app was designed in English and Spanish versions and was freely available as a download from the Apple App Store and Android Google Play App.

**Figure 1:**
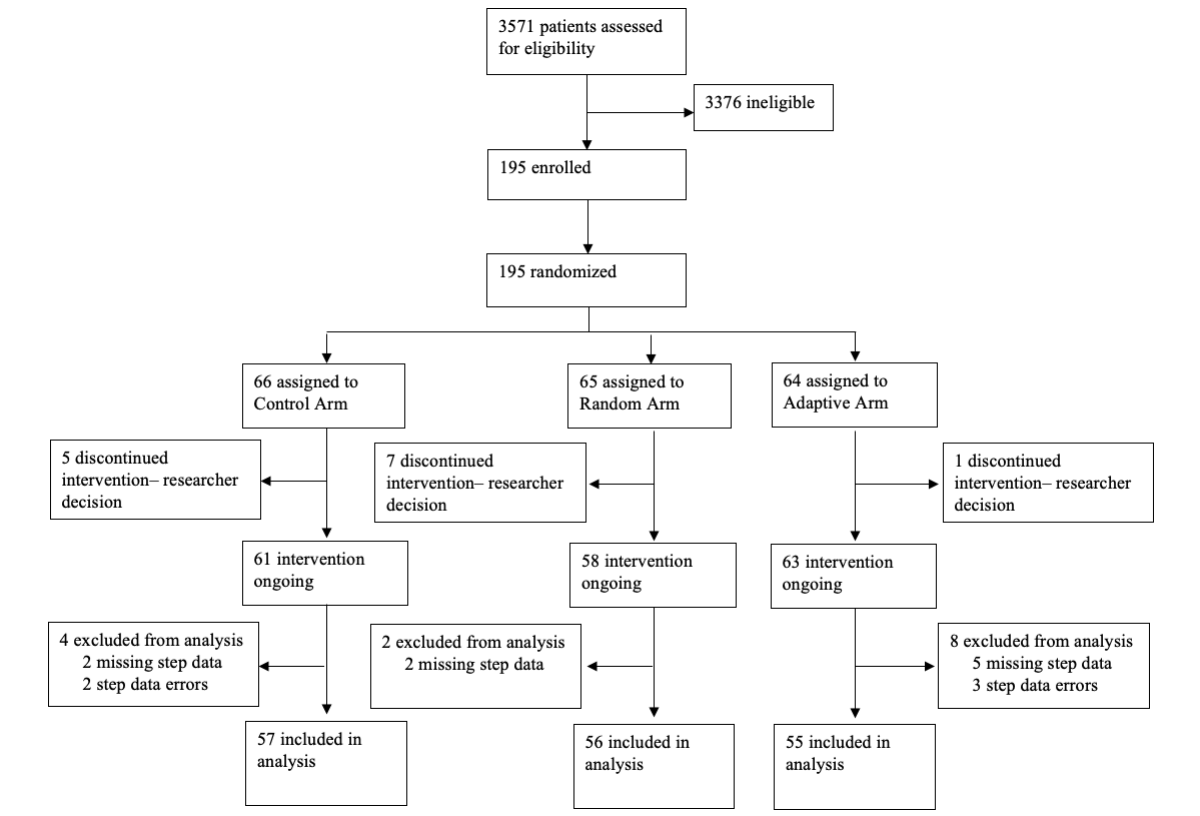
Diabetes and Mental Health Adaptive Notification Tracking and Evaluation (DIAMANTE) CONSORT (Consolidated Standards of Reporting Trials) diagram.

Figure 1 shows the different intervention groups during the trial period. The three comparator arms were (1) Control arm, who received only 1 weekly message inquiring about their mood over the past week, (2) Random messaging arm, who received daily randomly selected messages (1 feedback message on their step count [eg, “You walked 4000 steps yesterday”] and 1 motivational message about their health [eg, “Going for a walk can improve your mood and clear your mind]) from a preexisting bank of messages, and (3) Adaptive messaging arm, who received daily messages from the same feedback and motivational messaging banks, with message categories and times selected by a reinforcement learning algorithm. The Random and Adaptive arms also received the weekly mood message. In summary, the feedback messages were selected from 5 message types (step number, step number plus encouragement or reinforcement, relative [walked more or less relative to goal], relative [achieved or did not achieve goal], or no message), and motivation messages could be from 4 categories of messages based on the COM-B (capability, opportunity, motivation, and behavior) model (each with 18 message options in these categories, or a “no message” option). Messages were generated from behavioral science theoretical domains and final messages were selected based on user-centered design methods [19]; examples are shown in Table 1. Moreover, patients and end users provided feedback on the text messages, mobile app interface, and study protocols during user-centered design phases of the study [20].

**Table 1:** Feedback and motivational messages and algorithm message choices.

		Examples	Random (n=51), %	Adaptive (n=53), %	*P* value
**A: Feedback messages**					
	0. No feedback message		19.5	6.0	<.001
	1. Reaching goal	“Yesterday, you did not reach your goal”	19.9	20.8	.35
	2. Steps walked yesterday	“Yesterday, you walked 3824 steps”	20.5	28.1	<.001
	3. Walked more/less today than yesterday	“Yesterday, you walked more than your goal”	19.7	25.6	<.001
	4. Steps walked yesterday, plus a positive/negative motivational message	“You walked 8000 steps yesterday. Great job!”	20.4	19.5	.54
**B: Motivational messages**					
	0. No message	—^a^	24.3	9.3	<.001
	1. Capability, describes the physical and psychological benefits of walking and exercise	“Doing more physical activity can help reduce feelings of fatigue”	24.8	37.2	<.001
	2. Motivation, meant to increase self-confidence and the belief that one is capable of walking even in the face of challenges	“You have made changes to improve your health before, you can do it again”	25.6	19.3	<.001
	3. Opportunity, physical and social environment cues that make it more likely to engage in walking	“It there a local park you have been waiting to visit? Use it as an opportunity to get out of the house and do more steps!”	25.2	34.2	<.001

^a^Not applicable.

We developed the reinforcement learning algorithm (a type of machine learning) based on previous work [15]. The reinforcement learning algorithm had a “cold-start” beginning with a randomly selected message for each patient and used ongoing participant data for personalizing (1) the feedback message category selection, (2) the motivational message category selection, and (3) the timing of the message delivery daily (into 4 time periods from 8 AM to 8 PM)—upweighting messages and times that had a higher probability of increasing steps. As described previously, we used reinforcement learning algorithms for contextual multiarmed bandit problems [21], as these can maximize cumulative rewards in sequential decision tasks (as here, which sequences of messages optimally promote the highest step count in the upcoming day). We also used Thompson Sampling, a Bayesian method that can handle small amounts of data [22], which allowed the algorithm to continuously learn which feedback and motivational messages were effective for a user, based on contextual features like their previous step counts and which messages were sent previously, as well as participant data such as demographic and clinical characteristics (such as age, gender, and depressive symptoms scores collected at baseline). In summary, each morning within the adaptive intervention arm, the algorithms evaluated which categories of feedback and motivational messages would likely increase the step count for each participant in the upcoming day and at which time period the messages should be delivered to increase step count. The rates of algorithm message choices are shown in Table 1.

Researchers monitored the incoming step data to ensure that the system was collecting data to inform the algorithm and adaptive messages. Patients in all groups received reminders to open the app if no data were being transmitted from the DIAMANTE app to the HealthySMS system. An automated algorithm within HealthySMS triggered daily text messages to participants if the system did not receive step count data from the DIAMANTE app. The research team contacted the patients by phone for troubleshooting and to remind them to keep the app open in the background if patients’ phones were not transmitting data for more than 3 days (after the automated SMS reminders). In addition, patients in all groups could reply “STOP” or “PARAR” if they wished to stop receiving messages.

### Outcomes

Our primary outcome for the RCT, total change in daily step counts, was passively collected by a mobile phone application during the time that patients remain in the intervention (using Apple Healthkit, Google Fit, and pedometer data from participants’ phones). We anticipated some variability between methods of data collection but considered this to be a trade-off for greater generalizability and broader implementation. As specified in our Protocol paper [15], the a priori sample size was 276 patients to account for the 3 intervention arms and with 80% power and up to 15% (41/276) participant dropout. Similarly, prior to analysis, participants were required to have at least 28 days of step count data during the intervention in order to be included in the primary analysis, given that the algorithm needed sufficient step count data to be able to personalize content for each participant. Data quality was evaluated for all participants upon study exit (blinded by study arm), with removal of 3 participants with less than 28 days of step count data, 6 participants with no step count data, and 5 participants whose data signaled a major discrepancy (>25% of available daily step counts greater than 20,000 steps), as this may have indicated a flaw in the DIAMANTE app step count data extraction.

### Statistical Analysis

First, we reported on the overall demographics of the full sample and by intervention arm and overall step counts within the sample. Our per protocol primary analysis included all participants who had at least 28 days of steep count data and used longitudinal regression to determine the impact of the treatment arms on the change in step count for each participant. We analyzed changes in daily step count using a linear mixed-effects model (LMM) with an intervention variable (Adaptive arm as reference group, Control arm, and Random arm) and a time variable (day, Control × day, Random × day) as fixed effects and a random intercept for each participant. We plotted the raw step count data and the residuals to examine their distributions via histograms. As specified in our protocol, we also completed 1 a priori subanalysis: the impact of the intervention by recruitment sample (SFHN clinic-based recruitment vs web-based recruitment). We analyzed descriptive data in SPSS (version 28; IBM Corp) and the LMM analysis using the “lme4” package in R.

## Results

The research team recruited a total of 195 participants into the RCT between January 2020 and June 2022. The CONSORT (Consolidated Standards of Reporting Trials) diagram is shown in Figure 1. A total of 13 participants were dropped by the researchers due not installing or immediately deleting the DIAMANTE app, and another 14 were excluded because of insufficient (n=9) or incorrect (n=5) step count data. The final analytical sample included 168 participants, each of them is observed for 168 days. More than one-third of participants were recruited from the SFHN (63/168, 37.5%) and the remaining were recruited via the web (105/168, 62.5%). Baseline demographic characteristics stratified by the intervention arm are reported in Table 2. Overall, the sample participants were middle-aged (mean age 49, SD 12.1 years), predominantly female (104/168, 62%), and majority were English speakers (128/168, 76%). The sample was diverse in terms of race or ethnicity, education, and employment status. Finally, due to data transmission errors (eg, not having the app open) there was an average of 20 missing days of step data (SD 32.72) for study participants, and the average step count within the sample was 3221 steps at baseline and 3783 at follow-up. By arm, participants in the Control arm had an average of 3701 steps (SD 3054.53) on day 1 of the intervention and an average of 26 days of missing data (SD 36.91); participants in the Random arm had an average of 3471 steps on day 1 (SD 3274.78) and an average of 20 days of missing data (SD 32.54). Finally, Adaptive arm participants had an average of 2277 steps on day 1 (SD 2250.87) and an average of 14 days of missing data (SD 27.2).

**Table 2:** Baseline demographics.

		Control (n=57)	Random (n=56)	Adaptive (n=55)	Total (N=168)
Age (years), mean (SD)		51 (13.2)	49 (11.0)	48 (12.1)	49 (12.1)
**Sex, n (%)**					
	Female	35 (61)	38 (68)	31 (56)	104 (62)
	Male	20 (35)	18 (32)	23 (42)	61 (36)
**Language, n (%)**					
	English	42 (74)	44 (79)	42 (76)	128 (76)
	Spanish	15 (26)	12 (21)	13 (24)	40 (24)
**Race/ethnicity, n (%)**					
	Asian or Pacific Islander	4 (7)	4 (7)	4 (7)	12 (7)
	Black or African American	11 (19)	9 (16)	7 (13)	27 (16)
	White or Caucasian	14 (25)	17 (30)	19 (34)	50 (30)
	Latinx or Hispanic	22 (39)	20 (36)	22 (40)	64 (38)
	Multiracial/Ethnic	6 (11)	6 (11)	3 (6)	15 (9)
**Education, n (%)**					
	Some high school or less	11 (19)	10 (18)	8 (14)	29 (17)
	High school graduate	7 (12)	8 (14)	9 (16)	24 (14)
	Some college or technical school	9 (16)	17 (30)	18 (33)	44 (26)
	College graduate	24 (42)	16 (29)	15 (27)	55 (33)
	Graduate degree	6 (11)	5 (9)	5 (9)	16 (10)
**Employment, n (%)**					
	Full time (>35 hours/week)	19 (33)	19 (34)	22 (40)	60 (36)
	Part-time (<35 hours/week)	9 (16)	15 (27)	5 (9)	29 (17)
	Homemaker	2 (4)	3 (5)	6 (11)	11 (6)
	Unemployed	8 (14)	9 (16)	10 (18)	27 (16)
	Disabled/on disability	11 (19)	6 (11)	10 (18)	27 (16)
	Retired	7 (12)	4 (7)	1 (2)	12 (7)
**Depression scores**					
	PHQ-8^a^, mean (SD)	10.23 (6.54)	12.0 (6.67)	10.42 (6.0)	10.88 (6.42)

^a^PHQ-8: Patient Health Questionnaire-8.

**Figure 2:**
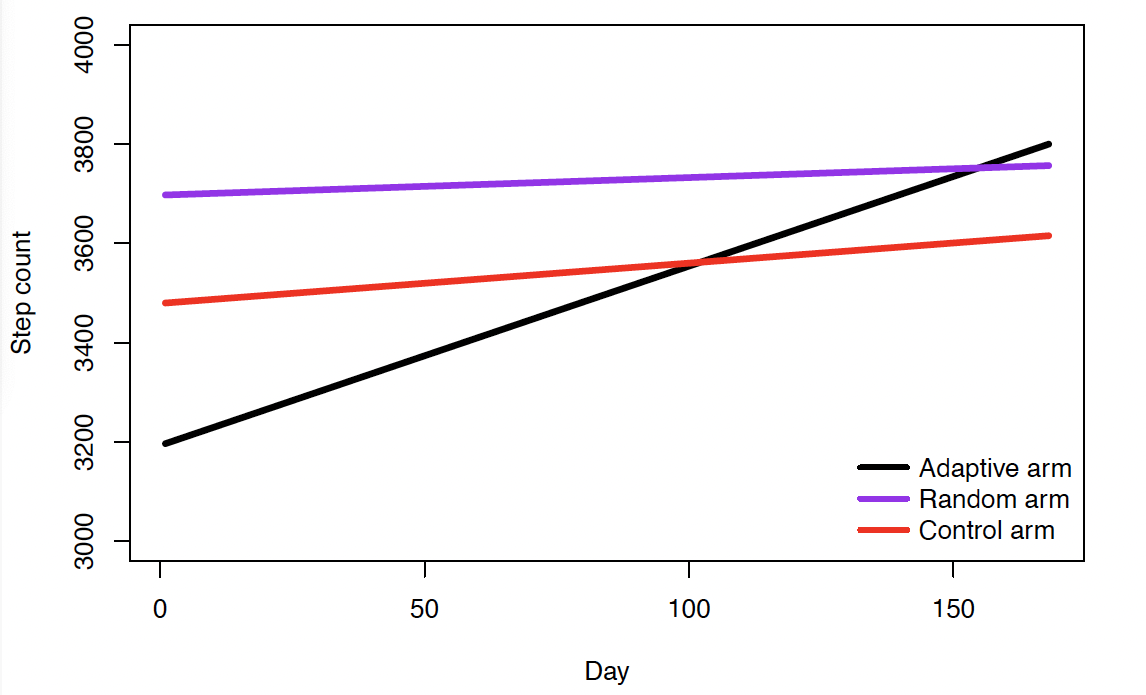
Linear trajectory of step count over 168 days of study.

The results of the primary LMM are shown in Table 3. The results indicate that on average, at the start of the intervention, participants in the Adaptive group had 3197 steps, the Control group had 3480 steps, and Random group had 3698 steps. The random intercept effects were estimated at 5,419,080.25 (SD 2237.39), indicating a large amount of variability in step count between participants. There were no statistically significant differences in baseline step count between intervention groups. For the Adaptive group there was a significant increase of 3.6 steps (*P*<.001) each day of the 24-week intervention; cumulatively and estimating for the 168-day time period, participants in the Adaptive group would have gained an average of 606 steps indicating a 19% increase in daily step count. The Control (*P*=.001) and Random (*P*<.001) groups had statistically significantly lower rates of change (albeit positive) in daily step count relative to Adaptive group. The Control group increased linearly by 0.81 steps per day for a total of 136 steps or an increase of 3.9% daily steps from day 1 to day 168 of the intervention. Finally, the Random group increased linearly by 0.35 steps per day for a total of 59 steps per day indicating an increase of 1.6% daily steps from day 1 to day 168 of the intervention (Figure 2).

**Table 3:** Linear mixed-effects model daily step count.

Parameter	Estimate	95% CI lower limit	95% CI upper limit	*P* value
Intercept (Adaptive)	3196.79	2568.03	3825.54	<.001
Control	283.32	–598.67	1165.32	.53
Random	500.92	–384.54	1386.36	.27
Day 1	3.61	2.45	4.78	<.001
Control (day 1)	–2.80	–4.47	–1.13	.001
Random (day 1)	–3.26	–4.92	–1.60	<.001

Based on our a priori subanalysis, the LMM results indicated statistically significant positive rates of change in daily step count for Adaptive group participants in both the SFHN subsample (average baseline steps 3278, daily rate of change 6.0 steps [*P*<.001], 30.6% increase in daily step count post intervention) and the web-based subsample (average baseline steps 3156, daily rate of change 2.3 steps (*P*=.002), 12.2% increase in daily step count post intervention). These results were in line with the overall analysis. However, in the SFHN sample the daily steps rate of change for the Random and Control groups was statistically significantly different and negative relative to the Adaptive group. Whereas for the web-based sample the daily rate of change for both the Random and Control groups was positive but not statistically significantly different than that of the Adaptive group.

## Discussion

Our study found that applying a reinforcement learning algorithm for personalizing text messaging interventions is an effective approach for increasing physical activity in a diverse sample of people with diabetes and depression. We found that significant step count increases over 6 months (168 days) in the adaptive, reinforcement learning intervention arm, as compared with participants who received randomly selected text message content (Random messaging arm) or only a weekly mood self-monitoring message (Control arm). We found that reinforcement learning algorithms can increase the effectiveness of a physical activity–based digital health intervention in a diverse, real-world sample with comorbid diabetes and depression symptoms. Our findings are particularly significant because most digital health studies in vulnerable populations to date have been pilot studies, and the use of machine learning methods for personalization is rarely applied to low-income and Spanish-speaking populations. These findings also support previous studies using a similar reinforcement learning algorithms [12].

Personalization (particularly those based on content that might be more meaningful for participants) has been one of the core challenges for the digital health field. Until now, many digital health studies show high dropout and low engagement of digital health interventions, also linked to a lack of personalization [23]. Here, we show that reinforcement learning holds promise for the future of digital health, especially given the rapid recent advancements in artificial intelligence. Our study importantly personalized the content using existing health behavior theory (COM-B), such as motivational content as opportunity cues for behavior change, which are likely important components for future machine learning interventions [24]. In this study, it appears that participants in the Adaptive messaging arm received messages that were more relevant in content as evidenced by the varying rates of message selection in the Adaptive arm versus the Random arm of the intervention. Furthermore, the real-world implementation of this study demonstrates the importance of establishing both efficacy and effectiveness of machine learning interventions [25].

We did find some differences in our primary step count outcome by recruitment source, with the clinic-based sample receiving more benefit from the intervention than the web-based recruitment sample (30% overall step count gain vs 12% overall step count gain among Adaptive group participants). The significant clinic-based sample findings are particularly of note, given that this population is primarily insured via Medicaid and receiving primary care within a public health care delivery system, which is reflected in the much higher proportion of Spanish speakers and individuals with lower educational attainment who typically do not have access to these types of interventions. There are various possible explanations for these differences that should be studied in future work including reaching a population that has less access to these types of interventions, better integration into care, or benefiting from co-design with similar populations.

A key strength of this study is the inclusion of a diverse sample in terms of race and ethnicity and language, education, and employment status. As machine learning algorithms continue to be developed and used in health interventions, it is crucial that the basis for these algorithmic approaches includes diverse and real-world samples so that algorithmic biases are minimized and their findings and potential benefits can be broadly shared. The results of this RCT provide valuable insights into the potential of adaptive messaging interventions to increase physical activity, especially in groups that are typically excluded from digital health research. If this intervention is widely disseminated and implemented, it can decrease health inequities by providing a type of personalized care for communities historically and presently marginalized.

Our study also highlights the need for further research to understand how digital health interventions can be tailored to different populations to maximize their effectiveness. Our future research will further investigate the parameters that influenced algorithmic decision-making, regarding the patterns of sending feedback messages, motivational messages, and timing of message delivery. It is also important to understand whether overall categories of messages were more impactful on behavior change and to understand how the algorithm can be refined to be more effective in achieving the goal of increasing physical activity in diverse samples.

Limitations of this study include challenges with data accuracy from phones. We chose to emphasize replicability in the real word and thus did not provide users with wearable devices or standardize the mobile phones that users had to have. This resulted in variability in data collection and some data collection errors. Furthermore, in order for these types of interventions to scale, we must maximize adoption and relevance (eg, use of devices that users already own) alongside precision (eg, “better” measurement using new devices).

Another limitation is that we did not specifically assess engagement with the messages. Similar to other text message interventions, we do not know whether or when people actually read messages; we only know when messages were sent. Finally, due to the unforeseen circumstances from the COVID-19 pandemic, recruitment number for our project was also less than our goal stated in the RCT protocol; however, our effect size was still large enough to be detected in the primary analysis.

The DIAMANTE intervention has shown that reinforcement learning algorithms can be used to improve the personalization of physical activity interventions in a diverse, multilingual sample with diabetes and depression symptoms. Moving forward, artificial intelligence and machine learning interventions will rapidly expand, and real-world studies targeting diverse end users are critical, especially when built upon co-design with end users.

## Appendix

Multimedia Appendix 1 CONSORT eHealth Checklist (v1.6.1).

## Abbreviations

COM-B: capability, opportunity, motivation, and behavior
CONSORT: Consolidated Standards of Reporting Trials
DIAMANTE: Diabetes and Mental Health Adaptive Notification Tracking and Evaluation
LMM: linear mixed-effects model
PHQ-8: Patient Health Questionnaire-8
RCT: randomized controlled trial
REDCap: Research Electronic Data Capture
SFHN: San Francisco Health Network
UCSF: University of California San Francisco

## Data Availability

The data sets generated during and/or analyzed during this study are available from the corresponding author on reasonable request.
